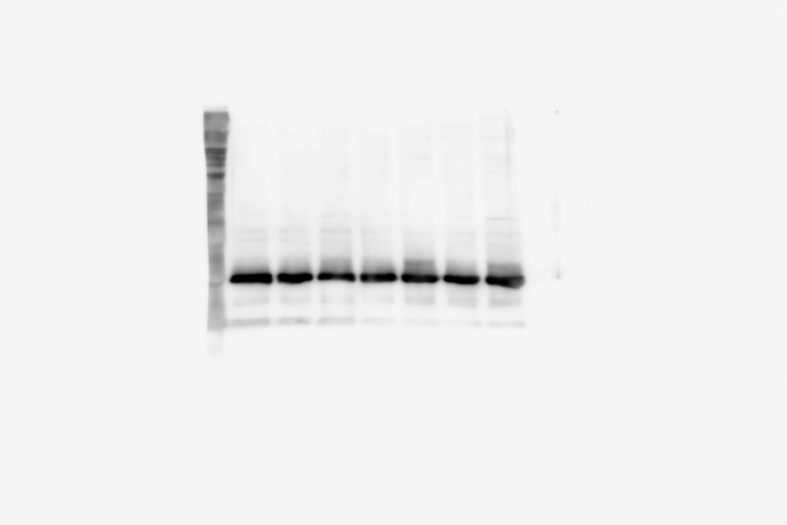# Involvement of activator protein-1 family members in β-catenin and p300 association on the genome of PANC-1 cells

**DOI:** 10.1016/j.heliyon.2022.e08890

**Published:** 2022-02-02

**Authors:** Tomomitsu Doi, Hironori Hojo, Shinsuke Ohba, Kunie Obayashi, Motoyoshi Endo, Toshimasa Ishizaki, Akira Katoh, Hiroyuki Kouji

**Affiliations:** a Department of Molecular Biology, School of Medicine, University of Occupational and Environmental Health, Japan, 1-1 Iseigaoka, Yahatanishi-ku, Kitakyushu, Fukuoka, 807-8555, Japan; b Center for Disease Biology and Integrative Medicine, Graduate School of Medicine, The University of Tokyo, Tokyo, 113-8656, Japan; c Department of Cell Biology, Institute of Biomedical Sciences, Nagasaki University, Nagasaki, 852-8588, Japan; d Department of Pharmacology, Faculty of Medicine, Oita University, 1-1 Idaigaoka, Hasama, Yufu, Oita, 879-5593, Japan; e Department of Clinical Pharmacology and Therapeutics, Faculty of Medicine, Oita University, 1-1 Idaigaoka, Hasama, Yufu, Oita, 879-5593, Japan; f Translational Chemical Biology Laboratory, Faculty of Medicine, Oita University, 1-1 Idaigaoka, Hasama, Yufu, Oita, 879-5593, Japan; g Oita University Institute of Advanced Medicine, Inc., 17-20, Higashi Kasuga-machi, Oita-city, Oita, 870-0037, Japan

**Keywords:** Wnt, β-catenin, Coactivator CREB-binding protein, p300, AP-1

## Abstract

Wnt/β-catenin is believed to regulate different sets of genes with different coactivators, cAMP response element-binding protein (CREB)-binding protein (CBP) or p300. However, the factors that determine which coactivators act on a particular promoter remain elusive. ICG-001 is a specific inhibitor for β-catenin/CBP but not for β-catenin/p300. By taking advantage of the action of ICG-001, we sought to investigate regulatory mechanisms underlying β-catenin coactivator usage in human pancreatic carcinoma PANC-1 cells through combinatorial analysis of chromatin immunoprecipitation-sequencing and RNA-sequencing. CBP and p300 preferentially bound to regions with the TCF motif alone and with both the TCF and AP-1 motifs, respectively. ICG-001 increased β-catenin binding to regions with both the TCF and AP-1 motifs, flanking the genes induced by ICG-001, concomitant with the increments of the p300 and AP-1 component c-JUN binding. Taken together, AP-1 possibly coordinates β-catenin coactivator usage in PANC-1 cells. These results would further our understanding of the canonical Wnt/β-catenin signaling divergence.

## 1 Introduction

The Wnt signal transduction pathway governs embryonic development throughout the animal kingdom (Clevers, 2006). Wnt proteins also play a major role in tissue homeostasis by controlling the proliferation and differentiation of tissue stem cells in multiple organs. Wnt pathway dysregulation leads to degenerative diseases and the development of cancer.

β-catenin is a key transcriptional mediator component in the canonical Wnt signaling (Clevers, 2006; Niehrs, 2012; Nusse and Clevers, 2017; Otero et al., 2004). The stability and activity of β-catenin are controlled by the destruction complex (DC). The ligation of the Wnt receptor Frizzled (FZD) with Wnt leads to the accumulation and translocation of β-catenin into the nucleus by suppressing DC activity. In the nucleus, β-catenin induces target gene transcription by forming a complex with DNA binding proteins, T-cell factor (TCF)/lymphoid enhancer factor family members (Lammi et al.). The loss of DC components leads to an aberrant stabilization of β-catenin and constitutive β-catenin target gene activation (Clevers, 2006; Niehrs, 2012; Nusse and Clevers, 2017). Adenomatous polyposis coli (APC), a component of DC, was first identified as a causal gene in familial adenomatous polyposis and often mutated in sporadic colorectal cancer (Kinzler et al., 1991; Kinzler and Vogelstein, 1996; Nishisho et al., 1991; Wood et al., 2007). In addition, mutations were found in the Wnt/β-catenin signaling pathway component genes *AXIN1*, *AXIN2*, *RNF43*, and *ZNRF3* in colorectal cancer, hepatocellular carcinoma, pancreatic cancer, and adrenocortical carcinoma (Assie et al., 2014; Lammi et al., 2004; Liu et al., 2000; Satoh et al., 2000; Wu et al., 2011). Therefore, attempts have been made for the therapeutic targeting of Wnt/β-catenin signaling but proven unsuccessful due to the pleiotropic nature of this pathway.

β-catenin recruits transcriptional coactivators cAMP response element-binding protein (CREB)-binding protein (CBP) or its homologous protein p300, just as well as other basal transcription factors, to activate target gene expression (Moon, 2005; Teo and Kahn, 2010). Though CBP and p300 have long been treated as functionally redundant proteins due to their sequence homology and similar expression patterns, a growing body of evidence suggested their unique roles (Kasper et al., 2006; Kung et al., 2000; Roth et al., 2003; Yamauchi et al., 2002). CBP and p300 have distinct roles in cell growth, differentiation, and embryonic development.

It is difficult to investigate the unique roles of CBP and p300 in Wnt/β-catenin signaling using a genetic approach, due to their complex interactomes (Kasper et al., 2006). Small chemical inhibitors for protein-protein interactions are potentially powerful tools to investigate the role of specific interactions of large hub molecules (Ballone et al., 2018). ICG-001 was identified as an inhibitor of the Wnt/β-catenin signaling in the screening using TCF/catenin consensus luciferase reporter system TopFlash (Emami et al., 2004; McMillan and Kahn, 2005). ICG-001 specifically binds to CBP, but not to p300, and blocks its interaction with β-catenin (Emami et al., 2004). It shifts the balance of coactivators toward p300/β-catenin (Emami et al., 2004; Ma et al., 2005). The specific inhibition of the interaction of the large molecule by ICG-001 allows analyzing the molecular mechanisms of the distinct roles of CBP/β-catenin from that of p300/β-catenin. Wnt signaling is involved in both stem cell potency maintenance and differentiation (Hari et al., 2002; Otero et al., 2004; Reya et al., 2001; Sato et al., 2004). This contradictory outcome must be mediated by different Wnt/β-catenin pathways. ICG-001 suppresses cell proliferation and induces differentiation suggesting the different roles between CBP and p300 in Wnt/β-catenin-mediated cell growth and differentiation (Banerjee et al., 2012; Hasegawa et al., 2012). The effect of ICG-001 on the β-catenin target gene expression reportedly varies in a target promoter-specific manner. Certain β-catenin target genes use exclusively CBP or p300, whereas other both CBP and p300 (Emami et al., 2004; Kahn, 2011; Ma et al., 2005). These contrasting actions on each promoter provide unique pharmacological features to ICG-001 such as tumor cell-specific cytotoxicity, induction of stem cell differentiation, and the reversion of tissue fibrosis (Banerjee et al., 2012; Chan et al., 2015; Emami et al., 2004; Gang et al., 2014; Hao et al., 2011; Henderson et al., 2010). However, the mechanism that determines the promoter response to ICG-001 is unknown.

In this study, we conducted chromatin immunoprecipitation and massively parallel sequencing (ChIP-seq) and RNA-sequencing (RNA-seq) to reveal the genome-scale action of β-catenin and coactivators CBP and p300 upon ICG-001 treatment. We further identified that ICG-001 increases p300 binding to β-catenin-associated regions with an activator protein-1 (AP-1) binding DNA motif through the induction of the AP-1 transcription factor family members in PANC-1 cells.

## 2 Results

### 2.1 Abrogation of β-catenin/CBP interaction shifted β-catenin-associated regions

**Figure 1:**
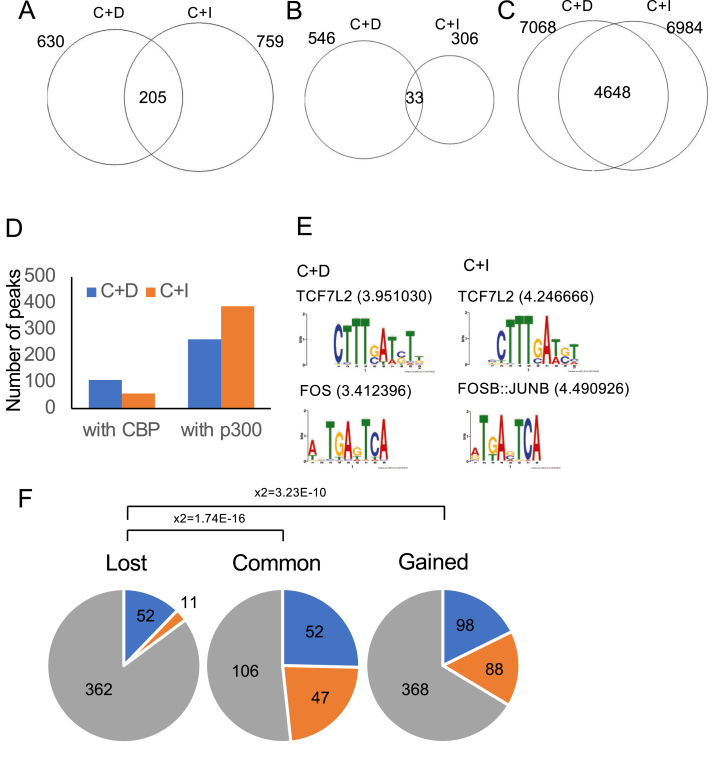
ICG-001 shifted β-catenin binding sites in PANC-1 cells (A, B, and C) Venn diagram showing the overlapping β-catenin- (A), CBP- (B), and p300- (C) associated regions on the genome in cells treated with CHIR-99021 + DMSO (C + D) and those treated with CHIR-99021+ICG-001 (C + I). (D) Number of β-catenin peaks overlapping with CBP or p300 in cells treated with DMSO or ICG-001. (E) Enriched motifs in β-catenin binding sites in cells treated with DMSO or ICG-001. Numbers indicate motif score. (F) Pie chart indicating the number of β-catenin-associated regions with binding motifs in common and lost or gained regions by ICG-001. Blue: with TCF motif only, Orange: with both TCF and AP-1, Gray: Others. Frequencies of regions with TCF and AP-1 motifs were compared with chi-square test.

To examine the effect of ICG-001 on the interaction of β-catenin with CBP and p300, we investigated the genome-wide distributions of β-catenin, CBP, and p300 in pancreatic carcinoma cells (PANC-1) using chromatin immunoprecipitation and massively parallel sequencing (ChIP-seq). PANC-1 cells were treated with Wnt/β-catenin activator CHIR-99021 along either with DMSO or ICG-001 (C + D or C + I). We identified 630 and 759 β-catenin-associated regions in cells treated with or without ICG-001, respectively (Figure 1A). Of these, 205 regions were common between these two profiles. As expected, most of CBP-associated regions were abrogated but shifted to different regions by the ICG-001 treatment (Figure 1B). In contrast, 66% of the p300-associated regions were unaffected by the ICG-001 treatment (Figure 1C). The intersection analysis with ChIP-seq peaks for β-catenin, CBP, and p300 showed that the peaks shared by β-catenin and CBP decreased in the ICG-treated group compared with the DMSO-treated group, whereas those by β-catenin and p300 increased in the ICG-treated group (Figure 1D). These results were consistent with the previously demonstrated ICG-001 effect through biochemical assays (Emami et al., 2004; Manegold et al., 2018): ICG-001 inhibited the interaction between β-catenin and CBP, increasing the interaction between β-catenin and p300. To determine the mode of DNA association of β-catenin, we conducted *de novo* motif analysis with sequences of β-catenin-associated regions. The TCF7L2 (TCF) and FOS:JUN (AP-1) motifs were significantly enriched in β-catenin-associated regions under both conditions (Figure 1E). In β-catenin-associated regions that were lost with ICG-001 (425 regions), 63 regions (14.8%) contained TCF motif and 11 regions (2.6%) contained both the TCF and AP-1 motifs (Figure 1F). In contrast, in β-catenin-associated regions that were gained with ICG-001 (554 regions), 186 regions (33.6%) contained TCF motif and 88 regions (15.9%) contained both the TCF and AP-1 motifs. 99 regions (48.3%) contained TCF motif and 47 regions (22.9%) contained both the TCF and AP-1 motifs in common regions (205 regions). In summary, these data showed higher enrichment of AP1 motifs in β-catenin-associated regions gained by ICG-001 as well as those unaffected by ICG-001, but not in those lost by ICG-001, suggesting potential involvement of AP-1 in the genomic redistribution of β-catenin in response to ICG-001.

### 2.2 β-catenin/p300, together with c-JUN, preferentially bound to regions harboring both TCF and AP-1 motifs

We examined the involvement of JUN family transcription factors on the genomic binding of β-catenin using RNA-seq data. Among the family members, only the JUN gene was upregulated by ICG-001 as reported previously (Teo et al., 2005) (Figure 2A). The time-course analysis showed that CHIR-99021 induced the expression of the JUN gene as early as 3 h after the treatment, and ICG-001 doubled the JUN gene expression as well as its coding protein c-JUN compared with CHIR-99021 alone (Figure 2B and C). Therefore, we examined the involvement of c-JUN at β-catenin binding sites by performing c-JUN ChIP-seq analysis. We identified 22732 peaks and 38741 peaks in C + D and C + I treated cells, respectively (Figure 2D).

**Figure 2:**
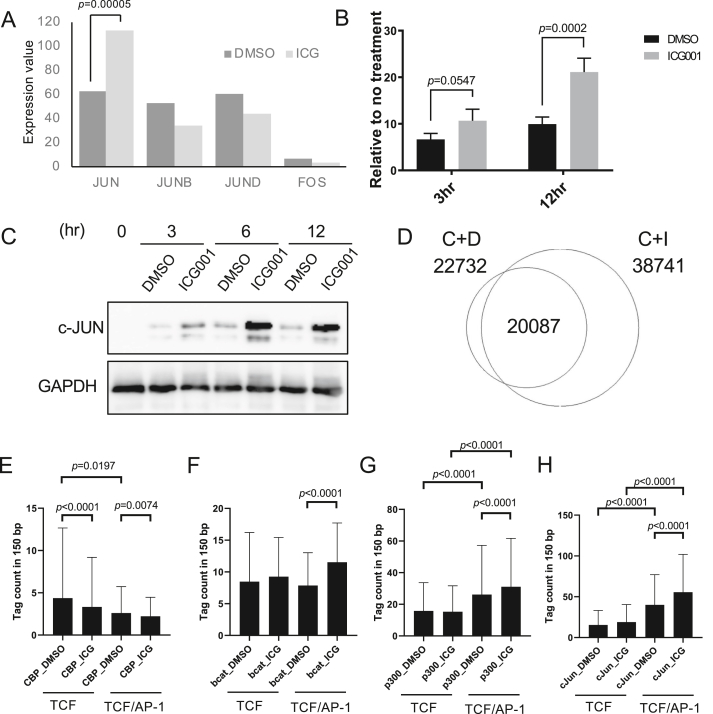
Expression of the JUN gene and genomic redistribution of β-catenin, CBP, p300, and c-JUN (A) Expression values of JUN, JUNB, JUND, and FOS in the RNA-seq data. n = 3. (B) qRT-PCR results of the JUN gene. PANC-1 cells were treated with CHIR and ICG-001 or CHIR alone for the indicated times. JUN expression was normalized with ACTB and relative values to non-treated PANC-1 cells were graphed. n = 3. (C) The protein level of c-JUN at indicated times after CHIR-99021 + DMSO or CHIR-99021+ICG-001 treatment. The protein level of GAPDH was used as a reference for total protein load. Original images are provided as Fig2_JUN and Fig2_GAPDH in supplementary materials. (D) Venn diagram showing overlapping regions bound by c-JUN in cells cultured with or without ICG-001. (E–H) Peak intensities in β-catenin-associated regions with the TCF motif alone and regions with the TCF and AP-1 motifs. TCF motif alone; n = 191, TCF and AP-1 motifs; n = 156. Error bars represent the standard deviation. The comparison between cells cultured with or without ICG-001 at the same peaks was analyzed via paired Student's *t*-test. The comparison between regions with TCF motif only and regions with TCF and AP-1 motifs were analyzed using unpaired Student's *t*-test.

To examine the role of the AP-1 motif in β-catenin/TCF signaling, we focused on β-catenin-associated regions with a TCF motif. We examined the peak intensities of β-catenin, CBP, p300, and c-JUN on β-catenin-associated regions with or without AP-1 motifs in the presence or absence of ICG-001. The analysis showed that the CBP peak intensity was decreased by the ICG-001 treatment under any conditions as demonstrated by a previous biochemical assay (Emami et al., 2004; Ma et al., 2005) (Figure 2E). The ICG-001 treatment increased the β-catenin peak intensity in the β-catenin-associated regions containing both TCF and AP-1 motifs but did not significantly alter the β-catenin peak intensity in the β-catenin-associated regions containing only the TCF motif (Figure 2F). Consistently, the p300 and c-JUN peak intensities were increased by the ICG-001 treatment in the β-catenin peaks containing both TCF and AP-1 motifs (Figure 2G and H). CBP peak intensities in the regions with the TCF motif alone were higher than those in the regions with both the TCF and AP-1 motifs (Figure 2E). In contrast, p300 and c-JUN peak intensities in the regions with the TCF motif alone were lower than those in the regions with the AP-1 motif (Figure 2G and H). These results suggest that p300 and CBP preferentially bind to the regions with the TCF motif alone or composite sites with the TCF and AP-1 motifs, respectively, and ICG-001 increases the binding of β-catenin, p300, and c-JUN only in the regions with the AP-1 motif.

**Figure 3:**
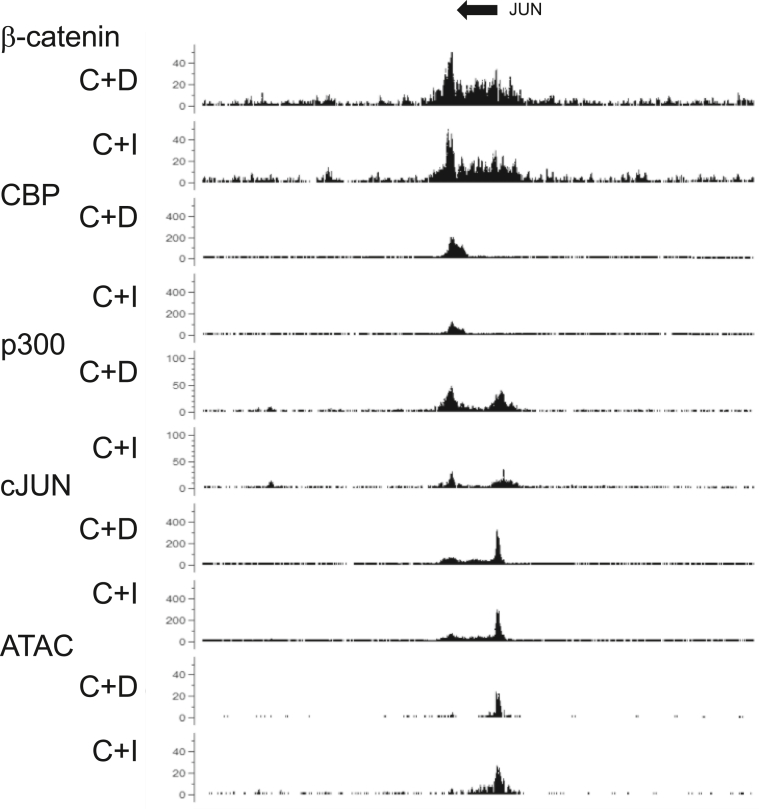
Screenshot of the CisGenome browser of the ChIP-seq peaks around the JUN gene. The arrow indicates the transcriptional direction. C + D: CHIR-99021 + DMSO, C + I: CHIR-99021+ICG-001.

To explore the effect of ICG-001 on the JUN gene transcription, we examined the binding of β-catenin, CBP, p300, c-JUN, and chromatin accessibility in the JUN gene. β-catenin, CBP, and p300 binding were found at the region downstream of the JUN gene, while p300 and c-JUN binding was found at the promoter of the JUN gene (Figure 3). Chromatin accessibility was increased at the promoter region by ICG-001 treatment.

### 2.3 β-catenin-bound regions with the TCF and AP-1 motifs were enriched around genes upregulated by the abrogation of β-catenin/CBP interaction

**Figure 4:**
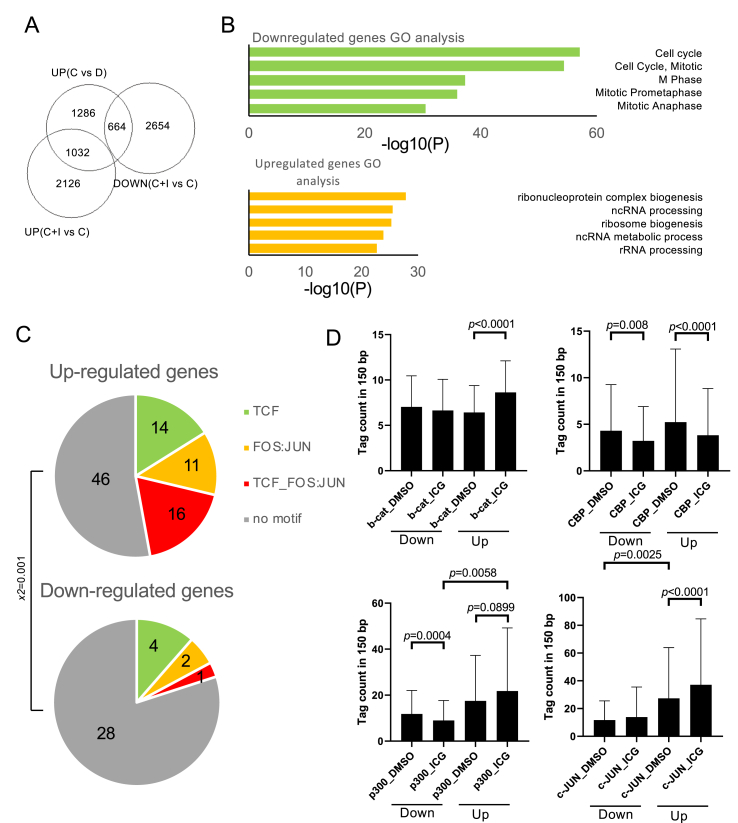
Regions with both TCF and AP-1 motifs were enriched near genes upregulated by ICG-001. (A) Venn diagram. UP (C vs. D) indicates genes upregulated by CHIR-99021 treatment vs. DMSO treatment. UP (C + I vs. C), DOWN (C + I, C) indicate up- or downregulated genes against CHIR-99021-treated cells. (B) GO analysis of DOWN (C + I vs. C) and UP (C + I vs. C) within UP (D vs. C). (C) Percentages of regions with TCF and FOS:JUN motifs near down- or upregulated genes. Frequencies of regions with TCF and AP-1 motifs were compared with chi-square test. (D) Peak intensities in β-catenin-associated regions near down- or upregulated genes. The comparison between cells cultured with or without ICG-001 at the same peaks was analyzed via paired Student's *t*-test. The comparison between regions near down- or upregulated genes were analyzed using unpaired Student's *t*-test.

To examine the effect of ICG-001 on the transcriptome, we conducted RNA-sequencing. The differential gene expression analysis revealed that 2982 genes were significantly upregulated in CHIR-99021 treated group, compared with the DMSO-treated group (Figure 4A, *p* < 0.05). In these differentially expressed genes, 664 and 1032 genes were down- and upregulated in cells treated with a combination of CHIR-99021 and ICG-001 (C + I), respectively, compared to cells treated with CHIR-99021 and DMSO (C + D). Gene ontology (GO) analysis showed that the top 5 GO terms enriched in downregulated genes were related to the cell cycle and GO terms enriched in upregulated genes were related to RNA biogenesis (Figure 4B). These results were consistent with the effect of ICG-001 on cancer cells as reported so far (Kahn, 2011).

To further integrate the transcriptome profiles with the various effects of β-catenin in the genome, we chose the nearest genes from β-catenin-binding regions as β-catenin putative target genes. Among the gene set, genes upregulated by the C + I treatment showed an increase in the proportion of both the AP1 motif and the TCF-AP1 composite motif in their neighboring β-catenin peaks compared with those downregulated by the C + I treatment (Figure 4C). In addition, β-catenin, as well as p300 and c-JUN, intensities were increased in the regions flanking genes upregulated by the ICG-001 treatment (Figure 4D). The p300 and c-JUN binding intensities at peaks associated with upregulated genes were higher than that in peaks associated with downregulated genes (Figure 4D). The CBP intensities under any conditions were reduced by the ICG-001 treatment (Figure 4D). This result suggests that a part of gene expression changes caused by ICG-001 is the result of β-catenin and AP-1 accumulation in the regions with an AP-1 motif caused due to an increase in JUN gene expression.

**Figure 5:**
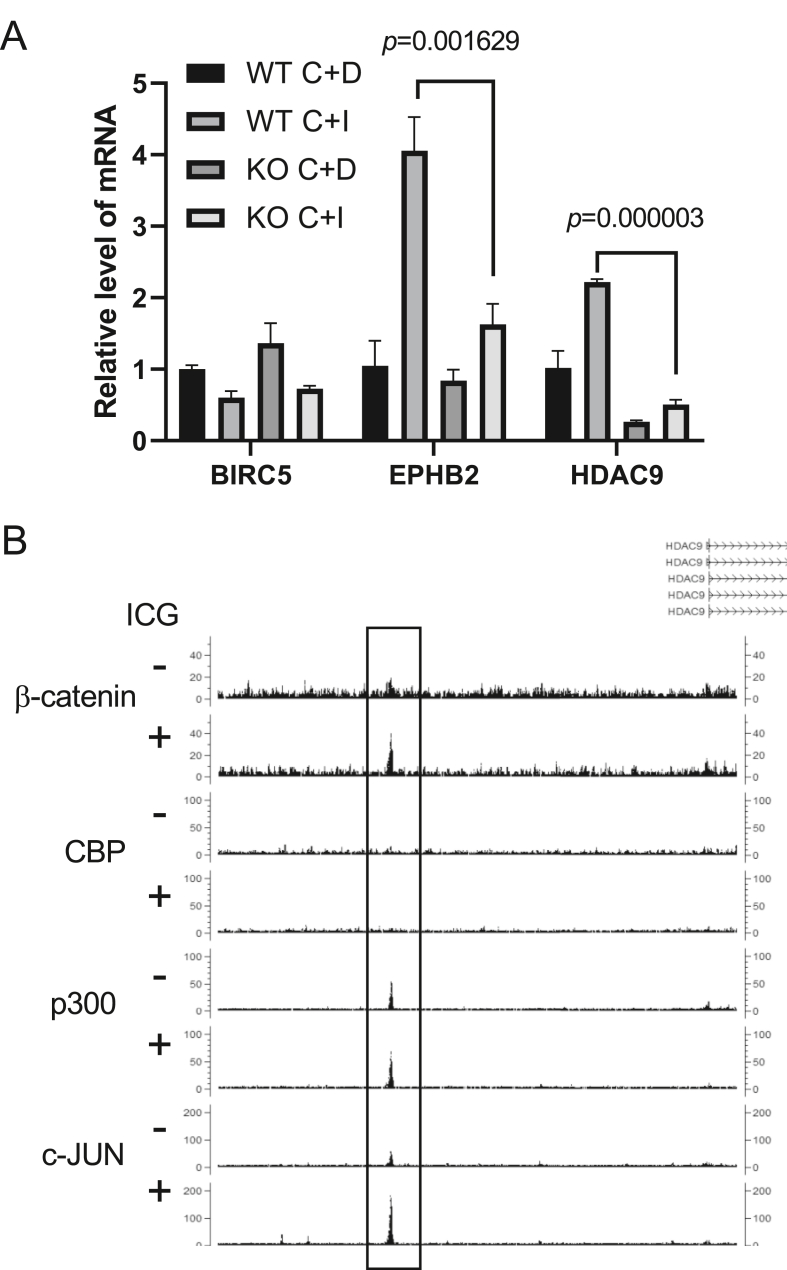
Effect of JUN knock-out (KO) on gene induction with ICG-001. (A) qPCR of mRNA expressions of BIRC5, EPHB2, and HDAC9 in wild-type and JUN KO PANC-1 cells treated with CHIR-99021 and with (C + I) or without (C + I) ICG-001. n = 3. Error bars represent the standard deviation. Expression differences between WT and KO cells were analyzed using unpaired Student's *t*-test. (B) Screenshot of the CisGenome browser demonstrating the ChIP-seq peaks of β-catenin, CBP, p300, and c-JUN upstream of HDAC9.

The c-JUN dependence of induction of these genes was validated by qRT-PCR in JUN knock-out (KO) PANC-1 cells. The effect of ICG-001 on transcriptions was confirmed by the suppression of BIRC5 and induction of EPHB2 as reported previously (Kahn, 2011). The induction of EPHB2 expression was observed to be c-JUN dependent (Figure 5A). Thirty-three genes upregulated by ICG-001 were associated with β-catenin peaks with both the TCF and AP-1 motifs. Among these genes, the expressions of HDAC9 and EPHB2 were decreased in JUN KO PANC-1 cells compared with those in wild-type PANC-1 cells (Figure 5A). Preferential binding of p300 over CBP was observed at a β-catenin-associated peak upstream of HDAC9 (Figure 5B).

## 3 Discussion

In this study, we explored the effect of ICG-001 on the genomic distribution of β-catenin and its binding partners CBP, p300, and c-JUN by ChIP-seq analysis. As expected from previous reports, ICG-001 decreased common peaks of β-catenin and CBP while it increased common peaks of β-catenin and p300 (Emami et al., 2004; Ma et al., 2005; Manegold et al., 2018). In addition, ICG-001 also induced genome-scale redistribution of β-catenin-associated regions suggesting that coactivator usage influences the selection of β-catenin target sites. Because the TCF-binding motif was most frequently observed at β-catenin peaks, TCF seems to be a major DNA binding partner of β-catenin as in the previous report (Franz et al., 2017; Schuijers et al., 2014). The AP-1 binding motif was a second major motif found at β-catenin-associated regions. A significant part of β-catenin peaks contained both the TCF and AP-1 motifs as reported in colon cancer cells (Bottomly et al., 2010). The fact that β-catenin-associated regions that were gained by ICG-001 treatment contained eight times more peaks with both the TCF and AP-1 motifs than the lost regions suggested the involvement of AP-1 in the β-catenin target site re-distributions. While ICG-001 treatment reduced CBP intensity at any conditions as expected from previous reports, p300 intensity was increased upon the ICG-001 treatment only in the β-catenin-associated regions with the AP-1 motif 24 h after treatment. The peak intensity of β-catenin was increased with increase of p300 and c-JUN peak intensities by ICG-001 treatment in the regions containing the AP-1 motif. p300 and c-JUN increment in the regions with an AP-1 motif could be explained by c-JUN protein induction that occurs after ICG-001 treatment. The choice of coactivators by a specific transcription factor with β-catenin appears to be dependent upon post-translational modifications of CBP and p300 (Lai et al., 2021; Ono et al., 2018). Preferential binding of CBP and p300 to the regions with TCF alone and the regions with the TCF and AP-1 motifs, respectively, raises the possibility that the AP-1 motif is a major determinant of post-translational modifications of CBP and p300 in the β-catenin-associated regions at least in PANC-1 cells. However, the mechanism of increment of β-catenin in these regions is not clear because β-catenin does not seem to bind directly to a complex of p300 and c-JUN in these regions. One possible mechanism is that p300 recruited by c-JUN through the AP-1 motif acetylates adjacent β-catenin at lysine 345 and thereby enhanced its affinity to TCF as reported previously (Levy et al., 2004).

As the regulatory sequences of a gene are assumed to be enriched in the vicinity of the given gene, β-catenin peaks were classified based on the expression of neighboring genes. There are few peaks with the AP-1 motif flanking genes induced by CHIR-99021 and suppressed by ICG-001. On the other hand, many peaks with the AP-1 motif, especially peaks with both the TCF and AP-1 motifs, were enriched at regions flanking genes induced by ICG-001. Peak intensities of β-catenin, p300, and c-JUN at the β-catenin peaks near upregulated genes increased with ICG-001 but not at peaks near downregulated genes, whereas peak intensities of CBP at any β-catenin peaks decreased. At β-catenin peaks with TCF motif alone, CBP seems to be a major coactivator for β-catenin target gene expression, as the p300 and c-JUN peak intensities at peaks near downregulated genes were lower than those near upregulated genes. Therefore, ICG-001 suppressed CBP-mediated target gene expression, whereas ICG-001 induced different target genes through recruitment of p300 and c-JUN at β-catenin-associated regions.

Herein, in addition to EPHB2, we identified HDAC9 as an ICG-001-c-JUN target. One of the problems with the therapeutic use of Wnt/β-catenin inhibitors is bone loss (Madan et al., 2018) because Wnt/β-catenin is involved in osteoblast differentiation from mesenchymal stem cells (Krishnan et al., 2006; Zhong et al., 2014). HDAC9 is involved in bone formation by suppressing osteoclast differentiation and promoting osteoblast differentiation (Jin et al., 2015; Li et al., 2015). ICG-001 may be able to solve this problem by inducing HDAC9 expression.

The functional interplay between β-catenin/TCF and AP-1 in the target gene expression has already been reported previously (Bottomly et al., 2010). Gene expression of c-JUN seems to be induced directly by β-catenin as Wnt/β-catenin signal inducer CHIR-99021 induced gene expression of the c-JUN and β-catenin binding site was observed immediately downstream of the JUN gene. ICG-001 further enhanced the expression of the JUN gene. The mechanism by which ICG-001 induces the JUN gene expression remains unclear. The gene-level expression of JUN is positively autoregulated by its product as supported by a previous report and c-JUN binding at the promoter in this study (Angel et al., 1988). ICG-001 reduced CBP binding at the β-catenin peak downstream of the gene and increased the accessibility of chromatin at the c-JUN peak of the promoter, suggesting that CBP in the downstream region may have a negative effect on the chromatin accessibility of the JUN promoter.

In summary, we propose that the AP-1 motif possibly determines the components of the β-catenin complex on the genome and the response to ICG-001 in the Wnt/β-catenin axis. Therefore, this property allows the specific expression inhibition of a part of β-catenin target genes with ICG-001. These findings lead to the elucidation of the gene expression switch mechanism by Wnt/β-catenin that controls different gene sets depending on cells and the development of therapeutics targeting the β-catenin target gene subset.

### 3.1 Limitations of the study

In this study, we were unable to examine the effect on the expression of genes distant from β-catenin binding regions as we analyzed the genes closest to these regions as putative targets. In future studies, investigating how cis-elements identified herein could influence the distant genes with information of comprehensive chromatin interactions would be essential.

## STAR methods

### Cell culture

PANC-1 cells were purchased from ATCC (CRL-1469, lot#6778038) and maintained in D-MEM supplemented with 10% fetal bovine serum and 100 U/ml penicillin and 100 μg/ml streptomycin. Confluent cells were stimulated by replacing the culture medium with a warmed medium containing 3 μM of CHIR-99021 (Chemscene LLC, CS-0181) and 30 μM of ICG-001 (synthesized in house) or the same volume of DMSO and cultured for 24 hours for ChIP-seq, ATAC-seq, and RNA-seq.

### ChIP-seq and ATAC-seq

ChIP-seq and ATAC-seq were performed as described previously (Ohba et al., 2015). Briefly, chromatin prepared from 1 × 107 PANC-1 cells was immunoprecipitated with anti-β-catenin (Cell signaling, D10A8), CBP (Abcam, ab2832), p300 (Abcam, ab14984), and c-JUN (Abcam, ab31419) antibodies. Libraries were prepared with ThruPLEX DNA-seq Kit (Takara-bio, R400674). For ATAC-seq, 50,000 nuclei were labeled with TD buffer (Illumina, 15027866) and Tn5 transposes (Illumina, 15027865). Libraries were sequenced with Illumina Hiseq X ten. Sequence reads were mapped to the human genome (hg19) with bowtie (Langmead et al., 2009). Binding peaks were determined with the CisGenome peak caller (Ji et al., 2008). Common peaks were determined by intersection with bedtools. De novo motif analysis was performed using the Gibbs motif sampler provided in the CisGenome package (Ji et al., 2008).

### RNA-seq

Total RNA was purified using the RNeasy Mini Kit (Qiagen, 74104). The libraries were prepared using the TruSeq standard mRNA library kit (Illumina, 20020594) and sequenced using NovaSeq 6000 (Illumina). The sequence reads were mapped to the human genome (hg19) with TopHat2 (Kim et al., 2013). The transcripts were assembled with Cufflinks (Trapnell et al., 2012). The differentially expressed genes were determined using Cuffdiff. GO analysis was conducted with Metascape (Zhou et al., 2019).

### Q-RT-PCR

Total RNA was purified with RNeasy Mini Kit (Qiagen, 74104). Complementary DNA was synthesized with ReverTra Ace qPCR RT Master Mix with gDNA Remover (Toyobo, FSQ-301). PCR was performed with ThunderBird SYBR qPCR mix (Toyobo, QPS-101) and Thermal cycler Dice (Takara-bio).

### Western Blot

Whole-cell lysates were prepared with a lysis buffer (PBS, 1% Triton-X100, Proteinase inhibitor Complete EDTA free (MERCK)). The concentration of extracted proteins was measured with the QuickStart protein assay reagent (Bio-Rad). Twenty micrograms of proteins were separated in 10% SDS-PAGE gel and transferred on Immobilon-P PVDF membrane (MECK). Membranes were blotted with anti-c-JUN antibody (Abcam, ab31419) or anti-β-actin antibody (BioLegend, 643808) with appropriate secondary antibody conjugated with horseradish peroxidase. Signals were developed with ECL prime (Cytiva, RPN2232).

### Establishment of JUN KO PANC-1 cells

LentiCRISPR v2 neo, psPAX2, and pCMV-VSV-G were obtained from Addgene. Sequences of guide RNAs were cloned into BsmBI sites of LentiCRISPR v2 neo vector. The lentivirus was rescued in 293T cells with psPAX2 and pCMV-VSV-G vectors. PANC-1 cells were infected with the virus and cloned under G418 selection. JUN KO PANC-1 clones were screened by western blotting for c-JUN.

## Declarations

### Author contribution statement

Tomomitsu Doi, Hironori Hojo: Conceived and designed the experiments; Wrote the paper.

Shinsuke Ohba: Conceived and designed the experiments.

Kunie Obayashi: Performed the experiments.

Motoyoshi Endo, Toshimasa Ishizaki, Hiroyuki Kouji: Analyzed and interpreted the data.

Akira Katoh: Contributed reagents, materials, analysis tools or data.

### Funding statement

This work was supported by 10.13039/501100001691JSPS KAKENHI Grant Number JP21K07972.

### Data availability statement

Data associated with this study has been deposited at GEO data base in NCBI.

### Declaration of interests statement

The authors declare no conflict of interest.

### Additional information

No additional information is available for this paper.

## Figures and Tables

**Fig2_JUN:**
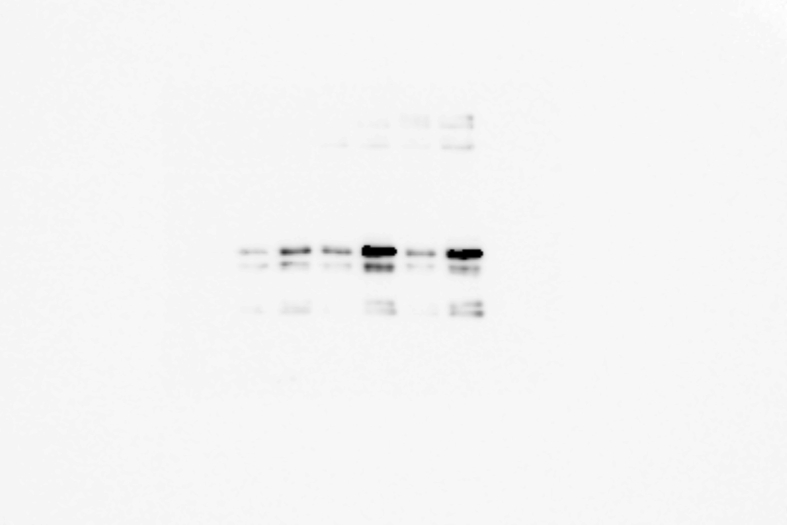


**Fig2_GAPDH:**